# Unicentric Castleman’s disease located between the aorta and inferior vena cava: A case report

**DOI:** 10.1177/2050313X19839532

**Published:** 2019-04-03

**Authors:** Masaki Murata, Oki Nagano, Go Hasegawa, Yohei Ikeda, Yuki Nakagawa, Yoshinobu Seki, Tsutomu Nishiyama, Yoshihiko Tomita

**Affiliations:** 1 Department of Urology, Uonuma Institute of Community Medicine, Niigata University Medical & Dental Hospital, Niigata, Japan; 2 Department of Hematology, Uonuma Institute of Community Medicine, Niigata University Medical & Dental Hospital, Niigata, Japan; 3 Department of Pathology, Uonuma Institute of Community Medicine, Niigata University Medical & Dental Hospital, Niigata, Japan; 4 Department of Diagnostic Radiology, Uonuma Institute of Community Medicine, Niigata University Medical & Dental Hospital, Niigata, Japan; 5 Departments of Urology and Molecular Oncology, Graduate School of Medical and Dental Sciences, Niigata University, Niigata, Japan

**Keywords:** Castleman’s disease, retroperitoneal tumor, unicentric

## Abstract

We report a 70-year-old woman diagnosed with unicentric Castleman’s disease with a contrast well-enhanced retroperitoneal tumor of 25 mm in diameter that located between the aorta and inferior vena cava. The imaging finding did not suggest any specific features, and no other lesion was detected. Laboratory examinations indicating malignant lymphoma such as soluble interleukin-2 were all negative. We resected the retroperitoneal tumor laparoscopically, and histopathological examination revealed hyaline vascular type Castleman’s disease. Although complete resection of hyaline vascular type unicentric Castleman’s disease results in a good prognosis, a late relapse has been reported and long-term follow-up is warranted.

## Introduction

Castleman’s disease (CD) is a rare lymphoproliferative disorder. The etiology remains unclear; however, several theories have been proposed recently. The previous report suggested the association with human herpesvirus 8 (HHV-8) and human immunodeficiency virus (HIV) infection.^1^,^2^ Moreover, chronic low-grade inflammation and interleukin-6 (IL-6) overproduction are thought to play a role in lymphoid hyperplasia and the pathogenesis of CD.^3^ CDs are categorized as unicentric and multicentric disease according to the anatomical distribution, and hyaline vascular (HV) type and plasma cell (PC) type according to the histological findings. Importantly, these subtypes are involved in different therapeutic strategies and prognosis.^1–3^ We present a surgically treated unicentric HV CD occurring between the aorta and inferior vena cava (IVC).

## A case report

**Figure 1.**
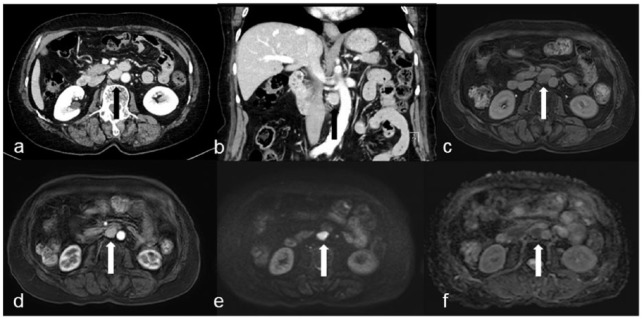
Abdominal CT shows a well-enhanced 25-mm retroperitoneal tumor that is located between the aorta and IVC (arrow): (a) axial CT image, (b) coronal CT image. MRI shows that the tumor is a high signal intensity on (c) T2-weighted image, (d) a well enhanced on T1-weighted image, (e) high signal intensity on diffusion-weighted image, and (f) low signal intensity on ADC-map.

**Figure 2.**
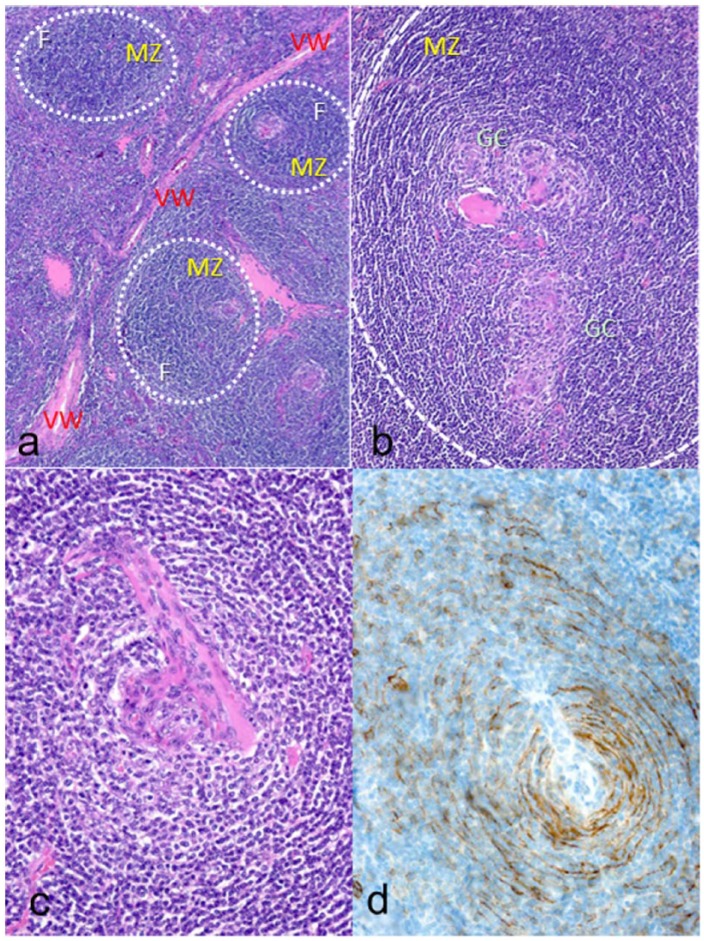
Pathological findings. Histopathologic findings show lymph follicles with expanding mantle zone. Intra-follicular stroma shows a marked proliferation of hyalinized vessels. The vessels penetrate germinal center to form a “lollipop” follicle, and follicular dendritic cells and small lymphocytes form concentric rings (“onion skin” appearance). These findings are consistent with those of hyaline vascular type Castleman’s disease. (a) Lymph follicles (F and white circle) with expanding mantle zone (MZ) and inter-follicular vascular proliferation with hyalinous thicken wall (VW). (b) Atrophic follicular germinal centers (GC) within a mantle zone. (c) Hyalinized vessel penetrating germinal center (“lollipop” appearance). (d) Follicular dendritic cell and small lymphocytes palisaded around germinal center (“onion skin” appearance). (a), (b), (c) Hematoxylin and eosin section and (d) CD23 staining.

A 70-year-old woman was referred to our hospital because of the abdominal discomfort lasting for 6 months. She had a history of appendectomy and depression, but there were no other comorbidities. There were no abnormalities on physical examination and blood test, including soluble interleukin-2 (sIL-2). HIV status test was also negative. Computed tomography (CT) scan showed a contrast well-enhanced retroperitoneal tumor of 25 mm in diameter that located between the aorta and IVC (Figure 1(a) and (b)). On magnetic resonance imaging (MRI), the mass had a high signal intensity on T2-weighted images and diffusion-weighted images, and contrast-enhanced homogeneously (Figure 1(c)–(f)). Our initial assumption that the tumor is paraganglioma was denied by the examinations; catecholamine levels in blood and urine were all normal, and ^123^I-metaiodobenzylguanidine (MIBG) scintigraphy was also negative. As excisional biopsy and treatment, we resected the tumor laparoscopically. Histopathologic examination showed lymph follicles with expanding mantle zone. Intra-follicular stroma showed a marked proliferation of hyalinized vessels. The vessels penetrated germinal center to form a “lollipop” follicle, and follicular dendritic cells and small lymphocytes form concentric rings (“onion skin” appearance; Figure 2). These findings were consistent with those of HV type CD. We underwent a whole-body MRI, and no other lesions were detected. Serum IL-6 level was within the normal range (<8 pg/mL). We diagnosed with unicentric Castleman’s disease (UCD) and decided to follow up without additional treatment. During the following 4 months, she had no recurrence of the disease.

## Discussion

CD is a rare atypical lymphoproliferative disorder that was first described in 1954.^4^ Although viral infection and chronic low-grade inflammation are involved in the onset of CD, the exact pathogenesis is still unclear. Histopathological evaluation is necessary for the definitive diagnosis because there are no disease-specific features in symptoms, a serum marker, and imaging.^1^ CD has three histologic subtypes: HV, PC, and mixed type.^1^,^2^

CDs are categorized as unicentric and multicentric disease according to the anatomical distribution. UCD is a localized and single mass without evidence of other clinical or radiological adenopathy.^3^ It occurs in various parts of the body: the chest (24%), neck (20%), abdomen (18%), retroperitoneum (14%), axillary region (13%), groin area (6%), and pelvic region (5%), respectively.^1^ Multicentric Castleman’s disease (MCD) is a systemic disorder that had similar histologic features to UCD, but involving multiple sites.^3^ In UCD, the HV type is dominant accounting for 78%, while the PC type accounts for 75% in MCD.^1^

These subtypes are directly related to the therapeutic approach and prognosis.^1–3^ The surgical approach for MCD does not necessarily improve its prognosis.^1–3^ The role of surgery is limited to obtain tissue for diagnosis and to relieve symptoms such as compression of vessels and airways, massive organomegaly, or bowel obstruction.^1^ Systemic therapy with steroids or anti-IL-6 monoclonal antibody has been used for MCD. However, the previous report suggests that the 3-year disease-free survival rate was 45.7% for MCD-PC patients.^2^

On the other hand, a surgical approach is a gold standard for the treatment of UCD. UCD has a favorable prognosis when achieved with complete surgical removal, and the cure rate is reported to be more than 90%.^1^ However, local recurrence has been reported to occur even 14 years after complete resection for UCD-HV; therefore, long-term follow-up is needed as it is in the present case.^5^

## Conclusion

We reported a case of UCD. Since the imaging finding does not suggest any specific features, histopathologic evaluation is necessary for the definite diagnosis of CD. In general, complete resection of UCD-HV has a better prognosis; however, late relapse has also been reported, indicating a need for long-term follow-up.
